# Chitinase Expression in *Listeria monocytogenes* Is Positively Regulated by the *Agr* System

**DOI:** 10.1371/journal.pone.0095385

**Published:** 2014-04-21

**Authors:** Dafni Katerina Paspaliari, Maria Storm Mollerup, Birgitte H. Kallipolitis, Hanne Ingmer, Marianne Halberg Larsen

**Affiliations:** 1 Department of Veterinary Disease Biology, Faculty of Health and Medical Sciences, University of Copenhagen, Frederiksberg C, Denmark; 2 Department of Biochemistry and Molecular Biology, University of Southern Denmark, Odense, Denmark; Technion-Israel Institute of Technology Haifa 32000 Israel, Israel

## Abstract

The food-borne pathogen *Listeria monocytogenes* encodes two chitinases, ChiA and ChiB, which allow the bacterium to hydrolyze chitin, the second most abundant polysaccharide in nature. Intriguingly, despite the absence of chitin in human and mammalian hosts, both of the chitinases have been deemed important for infection, through a mechanism that, at least in the case of ChiA, involves modulation of host immune responses. In this study, we show that the expression of the two chitinases is subject to regulation by the listerial *agr* system, a homologue of the *agr* quorum-sensing system of *Staphylococcus aureus*, that has so far been implicated in virulence and biofilm formation. We demonstrate that in addition to these roles, the listerial *agr* system is required for efficient chitin hydrolysis, as deletion of *agrD*, encoding the putative precursor of the *agr* autoinducer, dramatically decreased chitinolytic activity on agar plates. *Agr* was specifically induced in response to chitin addition in stationary phase and *agrD* was found to regulate the amount of *chiA*, but not *chiB*, transcripts. Although the transcript levels of *chiB* did not depend on *agrD*, the extracellular protein levels of both chitinases were reduced in the Δ*agrD* mutant. The regulatory effect of *agr* on *chiA* is potentially mediated through the small RNA LhrA, which we show here to be negatively regulated by *agr*. LhrA is in turn known to repress *chiA* translation by binding to the *chiA* transcript and interfering with ribosome recruitment. Our results highlight a previously unrecognized role of the *agr* system and suggest that autoinducer-based regulation of chitinolytic systems may be more commonplace than previously thought.

## Introduction

*Listeria monocytogenes* is a Gram-positive food-borne pathogen, and the causative agent of human listeriosis, a disease of varying severity that can prove fatal for immunosusceptible patient groups, such as pregnant women and the elderly.

*L. monocytogenes* is often isolated from marine environments, as well as from soil, where it lives as a saprophyte [1]–[3]. In these habitats, chitin, a polymer of *N*-acetylglucosamine (GlcNAc) and the second most abundant carbohydrate in nature, can constitute an important source of carbon and nitrogen [4]–[8]. Many bacteria autochthonous to chitin-rich environments have developed simple to complex chitinolytic systems, which allow them to degrade chitin and use it as a nutrient source [4], [8], [9]. Most importantly, chitin is broken down by chitinases, which are often assisted by lytic polysaccharide monooxygenases (LPMOs) that facilitate chitinase accessibility to the complex crystalline chitin structure [10], [11]. Interestingly, many human and animal pathogens are chitinolytic, and evidence has emerged that chitinases and LMPOs have an additional key role in these pathogens as virulence factors, which promote infection [12].

*L. monocytogenes* possesses a chitinolytic system that comprises two chitinases (ChiA and ChiB) and a putative LPMO (Lmo2467) [13], [14]. Both of the chitinases, but not Lmo2467, have been found to be important for colloidal chitin hydrolysis on agar plates [13]. ChiB contributes the most to the hydrolysis of this substrate and is the chitinase most induced during growth in soil [13], [15]. In contrast, ChiA has been identified as an important virulence factor associated with enhanced pathogenicity [16], [17]. Specifically, it was recently shown that ChiA helps *L. monocytogenes* suppress iNOS expression and thereby increases the rate of survival in the host [17].

During growth in rich laboratory media, the two chitinases are expressed only at background levels, and it is known that nutrient-poor conditions as well as the presence of an inducer are required for their full induction [18]. As is common with carbon utilization systems [19], stringent regulatory controls are in place to ensure chitinase expression only occurs under desired conditions. These include transcriptional dependence on the major global regulators σ^B^ and PrfA, as well as negative regulation by the small RNA LhrA, which we have previously shown to be negatively controlling translation of *chiA*, by binding to its mRNA and preventing ribosome recruitment [18], [20].

Another important regulatory machinery in *L. monocytogenes* is the accessory gene regulator (*agr*) system, which has orthologs in several Gram-positive bacteria, most notably *Staphylococcus aureus* [21]–[23]. Based on what is known about the function of the *agr* orthologs in the other bacteria, the listerial *agr* system has been proposed to be a quorum-sensing system composed of four main components (*agrBDCA*), co-transcribed in an operon [24]–[26]. According to this model, *agrD* encodes a propeptide, which is processed into the mature autoinducing peptide (AIP) and exported outside the cell by the transmembrane protein AgrB. When the extracellular concentration of the AIP reaches a certain threshold the system is activated through the two-component sensing system AgrC-AgrA and exerts regulatory effects on target substrates, as well as induces its own production (positive autoregulation).

The *agr* system of *L. monocytogenes* has been found to be important for key functions, such as biofilm formation and virulence [24]–[29]. However, microarray analyses suggested that it might play a far broader role in the bacterium, as they revealed a rather large *agr* regulon [26], [29]. Still, little is known about other roles of *agr*, or about its regulatory mechanism.

In this paper, we present a newly-identified role for the *agr* system in the regulation of the chitinolytic activity of *L. monocytogenes*. Additionally, we provide evidence that the levels of the sRNA LhrA are negatively regulated by *agr* and postulate that LhrA could be an effector of the system, mediating the regulatory effect of *agr* on *chiA*. Finally, we demonstrate that *agr* itself responds to the addition of chitin in the extracellular medium.

## Materials and Methods

### Bacterial strains and standard growth conditions

The *ΔagrD* mutant and its parental wild-type EGD-e strain were kindly provided by Colin Hill [26]. The chitinase deletion mutants and their parental EGD-e wild-type strain were obtained from Raghupathi *et al*. [30], and Dr. W. Goebel (Biozentrum, University of Würzburg, Germany), respectively. EGD-e strains from different laboratories may vary genetically. Therefore, mutants were always compared to their parental wild types, although when compared no differences were observed between the two wild-type parental strains.

Bacteria were routinely grown in brain heart infusion (BHI, Oxoid) aerobically at 37 °C, unless stated otherwise.

### Colloidal chitin preparation

5 g of chitin from shrimp shells (C9213, Sigma-Aldrich) were treated overnight with 50 mL 36–38% HCl. After treatment, the pH was adjusted to 8 by addition of NaOH. Subsequently, the mixture was centrifuged at 8228 × g for 5 min, and the chitin pellet was separated and washed seven times in ultrapure water.

### Chitinase assay

Chitin hydrolysis was assayed on LB (Oxoid) agar plates containing 6 mg/mL acid-hydrolyzed colloidal chitin. Specifically, 10 µL of overnight cultures were spotted on the plates and chitinase activity was assayed by measuring the diameters of the produced clearing zones after 4 days of incubation under aerobic conditions at 30 °C.

### Sample collection for total RNA extraction and beta-galactosidase assay

For sample collection, the cells were grown aerobically in LB at 30 °C and 190 rpm overnight. The next day, the cultures were diluted in LB supplemented with 0.05% glucose to an OD_600_ of 0.05, and thereafter grown at 30 °C and 190 rpm.

For estimation of *agrA* and chitinase gene transcription, the mutant and/or wild-type strains were grown until late-exponential phase (OD_600_ 0.7). At that point the cells were divided into two flasks, in one of which the cells were induced by the addition of colloidal chitin (see above) to a final concentration of 3.3 g/L. Samples for RNA were collected from both flasks (with and without chitin) 15 min and 2 h after induction, corresponding to late exponential and stationary (OD_600_ approximately 0.9) phase, respectively.

For estimation of *lhrA* transcription, the wild type and mutant samples were collected at mid-exponential and stationary phase (OD_600_ approximately 0.35 and 0.9). The same method was applied for the collection of samples for the beta-galactosidase assay, with the exception that the medium additionally contained kanamycin at a concentration of 50 µg/mL.

### Total RNA extraction

The cells were lysed with the aid of a FastPrep-24 Instrument (MP Biomedicals) for two rounds of 40 s at speed setting 6.0. Total RNA was extracted with the RNeasy mini kit (Qiagen), according to the manufacturer's directions. RNA concentration was measured in a Nanodrop 1000 spectrophotometer and the integrity of RNA was confirmed by visualizing the RNA samples on a 1% agarose gel.

### Northern blot analysis

For the northern blot analysis of *agrA* transcripts, 5 µg of RNA samples were denatured and separated on a 1% agarose gel containing 10 mg/mL ethidium bromide. After visualization under UV light (loading control), the RNA from the gel was transferred to a Hybond-N+ nylon membrane (GE Healthcare Life Sciences) by overnight capillary blotting. The radioactive probes were generated by PCR amplification and were subsequently ^32^P-radioactively-labeled using the Ready-to-Go DNA labeling Beads (GE Healthcare Life Sciences). Primers AgrA-F: CGAATGCCTACACATCAAGG and AgrA-R: CTTCACCACACCTTTTGTCG were used for the amplification of the *agrA* probe, while the primers for the *chiA* and *chiB* probes were the same as described in [18]. RNA hybridization was performed overnight at 65 °C in 0.5 M sodium phosphate buffer pH 7.2 containing 7% SDS. After washing in 20 mM sodium phosphate buffer pH 7.2 containing 1% SDS, the radioactive signal was detected with the aid of a Cyclone Plus Phosphor Imager (PerkinElmer) and analyzed with the accompanying OptiQuant software. Differences between the amounts of transcripts were considered relevant only if they exceeded 2-fold.

The northern blot analysis of *lhrA* was performed as described in [20].

### Beta-galactosidase assay

Previously constructed [20] fusions of the pTCV-lac vector with *lhrA* promoter fragments were introduced into the *ΔagrD* mutant and its parental EGD-e strain by means of electroporation [31]. The beta-galactosidase assay was performed as described in [32].

### Protein precipitation of bacterial supernatants

For sample collection of GlcNAc-induced cells, the cells were initially grown aerobically in LB at 30 °C and 190 rpm overnight. After the overnight incubation half of the cells were induced by dilution in LB (final OD_600_ of 0.05) supplemented with 1 g/L GlcNAc (Sigma-Aldrich). After overnight growth at 30 °C and 190 rpm, the OD_600_ of the uninduced cells was recorded. 9 mL of induced cells were spun down at 4629 × g at 0°C for 7 min. The supernatants were transferred to new tubes and the proteins were precipitated, by addition of 0.02% sodium deoxycholate (Sigma-Aldrich) and incubation for 30 min at 4°C, followed by addition of 10% trichloroacetic acid (Sigma-Aldrich) and overnight incubation at 4°C. The precipitated proteins were separated from the supernatant by centrifugation at 15000 × g for 15 min. Subsequently, the protein pellets were washed twice in 1.5 mL and 500 µL of ice cold 99% ethanol. Pellets were completely dried in a vacuum centrifuge and resuspended in 50 mM TrisHCl pH 8.0. The volumes of TrisHCl used for resuspension were calculated so that the samples would be normalized based on the recorded OD_600_ (50 µL of TrisHCl pH 8.0 per OD_600_ 1). 10 µL of the samples were used for the western blot analysis.

For sample collection of chitin-induced cells, the cells were initially grown aerobically in LB at 30 °C and 190 rpm overnight. The next day, the cultures were diluted in LB supplemented with 0.05% glucose to an OD_600_ of 0.05, and thereafter grown at 30 °C and 190 rpm. Upon reaching an OD_600_ of approximately 0.7, part of the cells were induced by transfer of 10 mL of culture to flasks containing 0.1 g of colloidal chitin (to reach a final concentration of 10 g/L). After overnight growth, the flasks were left to stand for 30 min, and the unhydrolyzed chitin fraction was separated from the culture by gravity. 9 mL of the culture were analyzed as described above for the GlcNAc-induced samples, with the exception that 5 µL and 1.25 µL instead of 10 µL were analyzed in the western blots of Fig. 5A and 5B, respectively. In parallel, the chitin fraction separated by gravity was washed twice in 1 mL 0.9% saline solution to remove bacterial cells. Subsequently, the chitin particles were separated by centrifugation at 16000 × g for 5 min. The pellet was resuspended in 20 µL of SDS-PAGE sample buffer, and boiled for 10 min at 99°C. 10 µL of the samples were used for western blot analysis.

### Western blot analysis

The samples were separated in 4–12% NuPAGE Bis-Tris gels (Invitrogen) under reducing conditions, and then transferred to a polyvinylidene difluoride (PVDF) membrane by use of the iBlot dry blotting system (Invitrogen). Western blot analysis was carried out with the use of the Western Breeze Chemiluminescent Kit-Anti-Rabbit (Invitrogen), according to the manufacturer's instructions. The anti-ChiA and anti-ChiB antibodies used for the analysis were raised in New Zealand white rabbits by Covalab. Purified N-terminally-HisTagged ChiA and ChiB proteins were used for immunization, respectively. Polyclonal serum was obtained after 53 days. Specificity was confirmed by comparison of blots of culture supernatants of wild-type EGD-e to blots of culture supernatants of Δ*chiA* and Δ*chiB* mutant strains (Fig. S1).

### Image processing

For presentation purposes, contrast and coloring were adjusted with Pixelmator.

## Results

### A Δ*agrD* mutant is impaired in chitin hydrolysis

**Figure 1:**
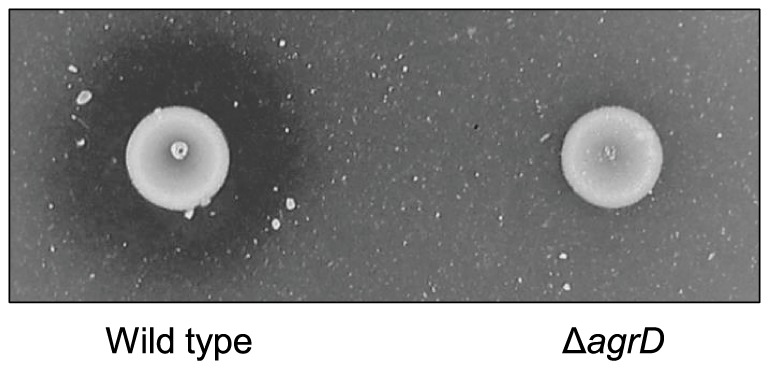
Chitinase activity of the wild-type EGD-e and its isogenic mutant lacking *agrD*. Bacterial cultures were spotted on LB-colloidal chitin agar plates and incubated at 30 °C for 3 days. The results presented here are representative of four independent experiments.

In *L. monocytogenes agrD* is predicted to encode the premature form of the autoinducing peptide of the *agr* system [26], [33]. In order to investigate the role of *agr* in chitin hydrolysis, we tested a mutant lacking *agrD* for chitinolytic activity on LB-chitin agar plates at 30 °C. We found the chitinolytic activity of the mutant to be clearly reduced when compared to that of the wild-type EGD-e (Fig. 1). This observation was supported by experiments in liquid cultures, where after overnight growth in LB supplemented with 0.05% glucose and 3.3 g/L colloidal chitin, the amount of unhydrolyzed chitin remaining in the supernatant was markedly larger in the case of the mutant (results not shown). No growth differences that could account for the observed phenotype were recorded between the two strains in LB medium supplemented with 0.05% glucose or on BHI and LB-chitin agar plates at 30 °C (data not shown). Similarly, the numbers of colony-forming units were comparable at selected points during growth in LB medium supplemented with 0.05% glucose and 3.3 g/L colloidal chitin.

### Deletion of *agrD* results in decreased transcripts of *chiA* but not of *chiB*

**Figure 2:**
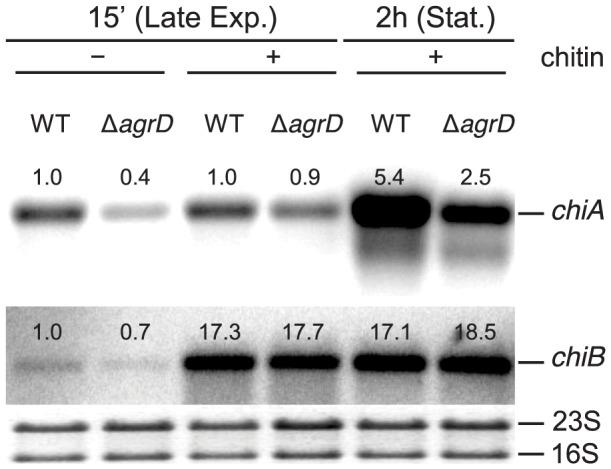
Northern blot analysis of *chiA* and *chiB* mRNA in the wild-type EGD-e and the Δ*agrD* mutant. Samples were taken 15-exponential and early stationary phase of growth, respectively. At the 15 min time point, RNA was also extracted from cultures grown without chitin and used as a reference. The numbers above the bands correspond to the relative fold change in relation to lane 1, i.e. to the transcript levels of wild-type bacteria in late exponential phase in medium without chitin. The loading control can be seen below each band. The results presented here are representative of a biological triplicate, collected and analyzed during the course of two independent experiments.

The impaired chitinolytic activity exhibited by the *agr* mutant could be the result of decreased expression of the chitinase genes *chiA* and/or *chiB*. To examine if *agrD* influences transcription of *chiA* and *chiB*, we compared the amounts of *chiA* and *chiB* mRNA produced in the wild-type and mutant strains. Transcripts were quantified 15 minutes and 2 hours following chitin addition to exponentially-growing cultures, at which time points the cells had reached late-exponential and stationary growth, respectively (Fig. 2).

In the absence of chitin, only low levels of *chiA* and *chiB* transcripts were observed in the late exponential phase (Fig. 2). In stationary phase, both transcripts are undetectable by northern blot [18] and were, therefore, not investigated. 15 minutes after chitin addition, *chiB* transcripts were induced to similarly high levels in the wild type and the Δ*agrD* mutant and remained as such until at least 2 h after induction. In contrast, *chiA* transcripts were only induced 2 h after chitin addition, when the cells had reached stationary phase, at which point the amount of *chiA* mRNA was approximately two-fold lower in the mutant lacking *agrD* compared to the wild type.

Therefore, it appears that *agrD* exerts a positive effect on *chiA*, but not *chiB*, expression at the transcript level.

### Deletion of *agrD* results in increased amounts of LhrA, a repressor of *chiA*

The product of the small RNA LhrA negatively regulates chitinase A, by binding to the *chiA* transcript and interfering with ribosome recruitment [20]. We hypothesized that the regulation of ChiA by *agr* might not be direct, but mediated through LhrA instead. To investigate that, we compared the expression of *lhrA* between the wild-type strain and the mutant lacking *agrD*, in mid-exponentially growing cells, as well as in cells that had reached stationary phase.

**Figure 3:**
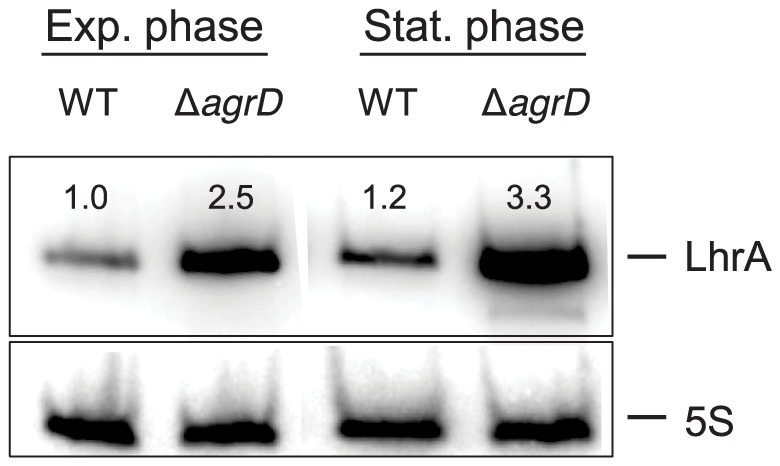
Northern blot analysis of *lhrA* transcripts in the wild-type EGD-e and the Δ*agrD* mutant. The two strains were grown at 30 °C in LB+0,05% glucose to mid-exponential and stationary phase. The numbers above the bands correspond to the relative fold change in relation to lane 1, i.e. to the transcript levels of wild-type bacteria in mid-exponential phase. The results are normalized to the 5S loading control, shown below each band and are representative of a biological triplicate, collected and analyzed during the course of two independent experiments.

Based on our results, deletion of *agrD* resulted in an approximately 2.5-fold increase in the levels of LhrA (Fig. 3), pointing to *agr* being a negative regulator of the small RNA. This supports our hypothesis that the decreased levels of *chiA* mRNA observed for the Δ*agrD* mutant were produced by the excessive LhrA amounts in the mutant background. Indeed, a decrease in *chiA* transcripts would be expected in the case of increased expression of LhrA, as the excessive LhrA would likely destabilize *chiA* transcripts by pairing with them and thereby promoting their degradation. Such a mechanism has been shown previously for another LhrA substrate [34]. It remains to be investigated whether the expected decrease in *chiA* transcripts in this case is enough to produce the *chiA* levels observed for the Δ*agrD* mutant, or whether additional regulatory interactions are involved.

### Investigation of *lhrA* transcription in the *agrD* mutant

To investigate whether LhrA regulation via *agr* occurs directly at the transcriptional level or rather post-transcriptionally, we compared *lhrA* transcription between the wild type and the Δ*agrD* mutant. To this end, we introduced two plasmids into the wild-type and mutant strains, carrying either the entire *lhrA* promoter ranging from position −157 to +71 relative to the transcriptional start site (LhrAp-157), or a truncated version of the promoter ranging from position −61 to +71 (LhrAp-61), fused to a promoter-less *lacZ* gene (for details see [20]).

**Figure 4:**
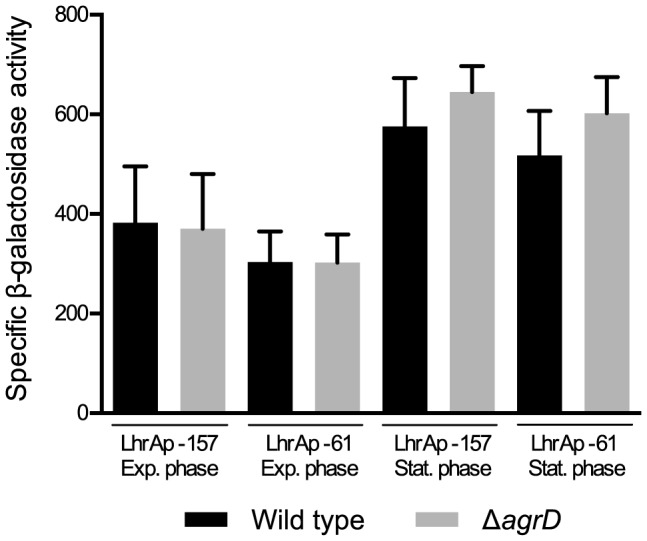
Effect of *agrD* deletion on transcription of *lhrA*. The wild type and *agrD* mutant containing transcriptional *lhrA-lacZ* fusions were grown at 30 °C in LB+0.05% glucose supplemented with kanamycin. Samples were collected in mid-exponential and stationary phase and β-galactosidase activity was measured. The results presented here are means of three independent experiments performed in duplicates.

*lhrA* transcription was quantified through measurements of specific beta-galactosidase activity in mid-exponential and stationary phase (Fig. 4). No significant differences were recorded between the full and truncated versions of the promoter for either strain, suggesting that there was no binding site for an *agr*-dependent transcriptional regulator between positions −157 and −61.

Upon comparison of *lhrA* transcription between the mutant and the wild-type strains, a small reproducible increase in transcription in the stationary phase was recorded for the mutant, but was not found to be statistically significant. We therefore propose that the regulation of LhrA by *agr* is exerted mainly at the post-transcriptional level.

### The extracellular levels of both ChiA and ChiB are reduced in the Δ*agrD* mutant

The deletion of *agrD* gave rise to an almost complete abolishment of chitinolytic activity on chitin agar plates (Fig. 1). However, comparison of the transcripts of *chiA* and *chiB* between the wild type and the mutant lacking *agrD* showed only limited effects of *agrD* on the levels of *chiA* (Fig. 2). Therefore, we hypothesized that *agrD* may have an additional impact on chitinase production at the post-transcriptional level. To confirm this, we carried out western blot analysis to compare the extracellular levels of secreted ChiA and ChiB after overnight incubation.

**Figure 5:**
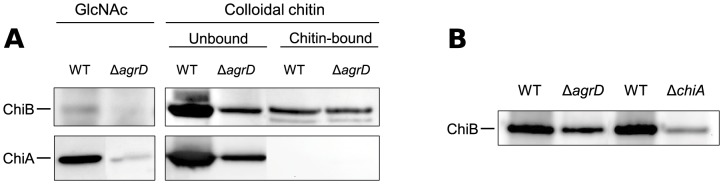
**A. Western blot analysis of culture supernatants of wild type EGD-e and the Δ*agrD* mutant.**The bacteria were grown overnight at 30 °C in LB+0.05% glucose, supplemented with either colloidal chitin or GlcNAc. In the case of colloidal chitin, two fractions are presented, representing the proteins remaining free in the supernatant (unbound), and those that remained bound to the chitin (chitin-bound). It should be noted that the loading of the two fractions was unequal. For comparison, the loaded amounts were such that the samples of the chitin-bound fraction represent a five times larger fraction of the total supernatant than the “unbound” samples. The results depicted here were reproduced in three independent experiments, except for the analysis of the chitin-bound proteins, which was confirmed in a biological duplicate. **B. Western blot analysis of culture supernatants of mutants lacking** ***chiA*** **and** ***agrD*** **, with their respective wild-type parental strains.** Samples were collected after overnight growth at 30 °C in LB+0.05% glucose, supplemented with colloidal chitin. Only the proteins remaining free in the supernatant and not bound to chitin are presented. The results were reproduced in a biological triplicate, collected and analyzed during the course of two independent experiments.

However, this type of analysis was complicated by the fact that it necessitated the addition of chitin as an inducer of chitinase expression, which, when added, bound part of the secreted chitinases (chitin-bound fraction of Fig. 5A). As this may influence the comparisons, we included both the bound and unbound chitinase fractions in the analysis (Fig. 5A). Although more unhydrolyzed chitin remained in the mutant cultures after the overnight incubation, we found the wild-type and mutant cultures to contain comparable amounts of chitin-bound ChiB, thus suggesting that chitin-binding should not be an important factor in this experimental setting. In support of that, we also found the amount of chitin-bound ChiB to comprise only a small fraction of the total amount of secreted ChiB. Binding of ChiA to chitin should not affect the results either, as we could practically detect no ChiA bound to chitin (Fig. 5A). A potential explanation for this is that ChiA lacks a chitin-binding domain, and thereby presumably has lower binding affinity towards colloidal chitin.

After the overnight incubation, both ChiA and ChiB could be detected unbound in the supernatant fractions in high amounts, but their levels were lower in the Δ*agrD* mutant compared to the wild-type strain (Fig. 5A). This suggests that *agrD* ultimately affects production and secretion of both chitinases. This should be the result of post-transcriptional regulation, at least in the case of ChiB, where no transcriptional difference was observed (Fig. 2).

In order to confirm the results with a soluble chitinase inducer we repeated the experiment with GlcNAC, which, in contrast to chitin, induces expression of only *chiA* and not *chiB* [18]. In support of the previous experiments, we found the amount of ChiA in the supernatant to be clearly reduced in the mutant lacking *agrD* (Fig. 5A). As *chiB* is not induced by GlcNAc, the overall levels of this chitinase in the supernatant were very low, thus hindering the comparison between the wild type and mutant. Nevertheless, there appeared to be a small decrease in the amount of secreted ChiB in the absence of *agrD*.

Taken together, our results show that deletion of *agrD* ultimately decreases production of both ChiA and ChiB.

### Deletion of *chiA* affects the amount of secreted ChiB

We have previously shown that although ChiB appears to be the main chitinase involved in colloidal chitin hydrolysis, a Δ*chiA* mutant strain is also severely impaired in chitin hydrolysis on chitin agar plates [13]. The level of difference appears to be disproportionate to the expected contribution of the deletion of *chiA* alone. Similarly, in our present study, deletion of *agrD* greatly affected the extracellular levels of ChiB, although no transcriptional effect was recorded. These two observations raised the question whether the reduced levels of ChiB observed for the *agr* mutant could be the result of the decreased production of ChiA caused by the *agr* deletion, rather than a direct result of the deletion itself. To investigate this possibility, we tested a Δ*chiA* mutant strain for impaired production of ChiB.

ChiB production was compared with the aid of western blotting, carried out as described above; however, only the free, non-chitin-bound protein fraction was analyzed. As can be seen in Fig. 5B, the levels of extracellular ChiB were greatly decreased in the Δ*chiA* mutant, confirming our assumption that *chiA* influences production of ChiB. No such effect was observed on the ChiA levels when the wild-type was compared to a Δ*chiB* mutant (Figure S1A). These observations suggest that, indeed, the reduced levels of ChiB observed for the Δ*agrD* mutant may be related to the decreased ChiA production in the mutant. However, this should be further confirmed, as our results do not disprove the existence of *chiA*-independent but *agr*-dependent post-transcriptional regulation. In addition, the reduced levels of ChiB in the two mutants should be confirmed in an assay using a soluble inducer that does not bind ChiB, instead of colloidal chitin.

### *Agr* is specifically induced by chitin in stationary phase

**Figure 6:**
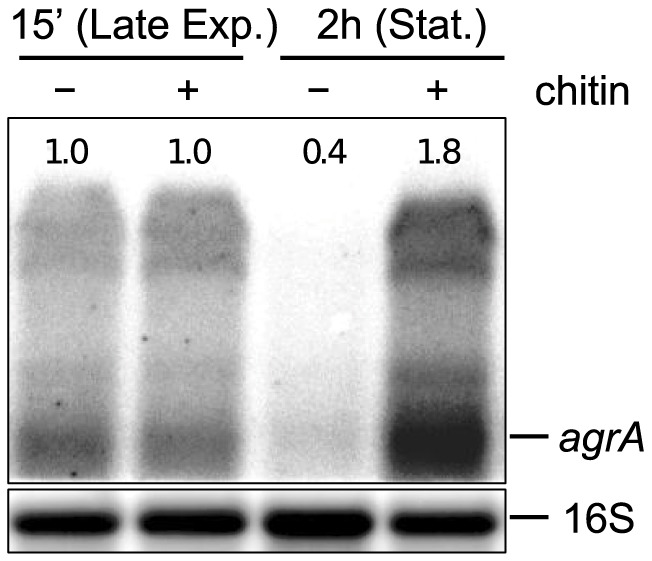
Northern blot analysis of *agrA* mRNA in the wild-type EGD-e strain in response to chitin addition. Samples were taken 15-exponential and early stationary phase of growth, respectively. The numbers above the bands correspond to the relative fold change in relation to lane 1, i.e. to the *agrA* transcript level 15 min after induction in medium without chitin. The loading control, probed for 16S RNA, can be seen below each band. The results presented here were reproduced in three independent experiments.

The observation that the *agr* system participates in the regulation of the chitinases prompted us to investigate how *agr* itself responds to chitin induction. To examine that, we looked for changes in the *agr* transcripts following chitin addition, with the aid of northern blot analysis (Fig. 6).

As a measure of the operon transcription, we quantified the transcripts of *agrA* 15 min and 2 h after chitin addition, which correspond to late exponential and stationary phase, respectively. Based on previous studies [24], [25], [35], *agrA* transcripts exist both as part of the whole operon in one long transcript, as well as in smaller fractions, likely resulting from degradation and further processing of the long transcript. In agreement with that, upon probing with the *agrA*-specific probe we identified two bands, likely corresponding to the mRNA of the whole operon, and the individual *agrA* transcripts (Fig. 6)

From the northern blot analysis it can be seen that in the absence of chitin *agr* transcription decreased in stationary phase compared to late exponential. A similar decrease in *agr* transcription in stationary phase has been reported previously for *Clostridium botulinum agrBD1* and *agrBD2* [36], and was also recorded for *L. monocytogenes* in luminescence reporter experiments for cells growing in LB at 37 °C [37]. Nevertheless, it was not noted in other studies investigating *agr* transcription in BHI at 37 °C [24], [25]. The reasons for this discrepancy are not clear, but may be related to differences in the conditions and the time-points of the measurements.

15 min after chitin addition, in late exponential phase, the levels of *agrA* appeared to be unchanged compared to the uninduced state. However, a clear upregulation in the presence of chitin was observed 2 h after induction, when the cells had reached stationary phase. A similar induction in stationary phase was observed when beta-chitin, instead of colloidal chitin, was used for the induction (results not shown). These results suggest that *agr* responds, directly or indirectly, to the presence of chitin in stationary phase, which strengthens the notion that *agr* is a regulatory component controlling the chitinolytic system of *L. monocytogenes*.

## Discussion

Numerous bacterial organisms are chitinolytic and produce chitinases that aid them in nutrient acquisition, as well as in virulence [4], [8], [12], [16], [38]. However, chitinase production is not constant throughout bacterial growth. Rather, it occurs mostly within a narrow window of nutrient limitation under the presence of an inducer, and is considered to be subject to stringent regulatory controls [5], [18], [39], [40].

In *L. monocytogenes* the regulatory mechanisms governing chitinase regulation are proving to be extremely complex, and include the central regulators PrfA and σ^B^, as well as the sRNA LhrA [18], [20]. In this study, we identified the *agr* system of *L. monocytogenes* as being additionally involved in the regulation of the chitinolytic system. Specifically, we found that deletion of *agrD*, the presumed precursor of the *agr* autoinducing peptide [26], [33], dramatically decreased the chitinolytic activity of the bacterium by interfering with the production and secretion of both listerial chitinases.

A moderate effect of the deletion was already seen on the transcript levels of *chiA*, but not of *chiB*. The observed reduction in the transcript levels of *chiA* is in agreement with a previous study by Riedel and colleagues [26], who recorded a similar effect in a microarray setting, albeit under different conditions. However, this effect was not seen in a microarray study by Garmyn and colleagues [29], who studied a mutant lacking *agrA*. This discrepancy may be a result of the conditions used for the microarray assay, which involved rich medium, 37 °C and no chitinase inducer, i.e. conditions under which chitinase transcription is normally very low [18]. No transcriptional effect was described for *chiB* in either of the two microarray studies, which is in accordance with our results.

Despite the lack of an effect at the transcript level, we found the extracellular levels of ChiB to also be reduced in the Δ*agrD* mutant, suggesting a post-transcriptional effect. This may be related to the altered expression of *chiA* in the mutant, as the deletion of *chiA* appears to cause a decrease in the production of ChiB. The exact nature of the post-transcriptional effect on *chiB* remains unknown, but mechanisms such as modulation of translation, protein stability and/or secretion could be involved.

Interestingly, we also found *agr* itself to be induced upon chitin addition in stationary phase, in a manner similar to that seen for *chiA*.

In *S. aureus*, *agr*-based regulation is mainly mediated through the sRNA RNAIII that acts as an effector for the system [21]. However, in *Listeria* no sRNA has been identified in connection with the *agr* system so far. The recent recognition of the sRNA LhrA as a negative regulator of *chiA* [20] prompted us to investigate whether it could be an intermediate component, mediating, at least partially, the response between *agr* and *chiA*. In support of this hypothesis, we found *agrD* to be a negative regulator of LhrA. This correlates with the specific decrease in the transcripts of *chiA*, but not *chiB*, that we saw in the case of Δ*agrD* mutant, given that LhrA has been found to exert a negative effect on the levels of the *chiA* mRNA [20]. Interestingly, in agreement with our results, Garmyn et al. [29] found *agrA* to repress transcription of *lmo2257*, which is an open reading frame overlapping with the *lhrA* gene.

Although this finding implicates LhrA in the *agr* response, LhrA does not appear to be an effector of *agr* similar to the staphylococcal RNAIII, as, in contrast to RNAIII [21], we found that LhrA is mainly regulated by *agrD* at the post-transcriptional level. The mechanism of regulation could involve decreased LhrA stability through the action of *agr*.

The regulatory effect of *agr* on LhrA implies that at least part of the regulation exerted by *agr* on *chiA* is through LhrA. However, due to the large number of genes that are under *agr* regulation in *L. monocytogenes*, the regulation of the chitinases via *agr* is likely mediated at different levels, and extends further than LhrA. For example, PrfA, which is itself a regulator of the chitinases, is deregulated in the Δ*agrD* mutant background [18], [26].

The implication of the *agr* system in chitin response is of great interest, as so far relatively little is known about the targets and role of *agr* in *Listeria*. Although not directly proven in *Listeria*, the system has been baptized as ”quorum-sensing” [26], given that *agr* is a recognized quorum-sensing (QS) system in other bacteria [23]. However, the discovery of environmental cues to which *agr* responds, such as temperature, has led other authors to propose that *agr* should not be viewed as a QS system in the strict sense, but should be considered to have pleiotropic effects of relevance to the environmental lifestyle of the bacterium [29], [41]. Our discovery that *agr* regulates chitinolytic activity further supports this view.

The importance of QS-like systems in non-strictly population-dependent responses has been underscored before, and has led to the formulation of different terminologies and theories regarding the role and evolution of such systems in bacteria [42], [43]. Overall, autoinducing molecules have been proposed to be secreted as proxies that estimate the efficiency of producing extracellular diffusible effectors, by taking into account parameters such as cell density, diffusion limitations and spatial distribution [43]–[45]. According to these models, QS systems then guide the production of enzymes only when autoinducer recovery, as a measure of enzyme recovery, exceeds a certain threshold. Enzymes regulated in this way have been suggested to include bacterial chitinases [46], and, therefore, similar arrangements might also be relevant for *Listeria*. In soil habitats where *Listeria* is autochthonous [3], competition for the scarce resources may pressure for the production of energy-costly systems, such as the chitinolytic system, only at opportune moments [6], [46], [47]. Using an autoinducer for the sensing, instead of the chitinase macromolecule, could offer the advantage of being much more economical in production, while only needing to be produced in low numbers, as it can provide a fast response due to the positive autoregulatory feedback [45].

In support of these views, links between QS systems and chitin utilization have been reported in other bacteria as well. In *Pseudomonas aeruginosa*, the chitinase ChiC and the chitin-binding protein CbpD have been identified as part of the QS regulon [48], [49]. In *Chromobacterium violaceum* the production of a set of chitinases is positively controlled by the QS endogenous *N*-hexanoyl-L-homoserine lactone (HHL) signal [50]. In contrast, negative regulation of chitinase activity by the QS system has been reported for *Vibrio harveyi* [51]. Finally, there is also evidence for QS-based control of chitinase activity in *Serratia proteamaculans* [52], as well as in *Serratia marsescens* [53], a model organism for the study of chitinolytic systems. Nevertheless, to our knowledge, regulation of chitinase production by QS systems had only been reported in Gram-negative bacteria so far, whose QS systems vary significantly and structurally from the Gram-positive ones. The discovery that the Gram-positive listerial *agr* system is also involved in chitin degradation suggests that this mechanism may be more intrinsic and widespread than previously recognized. In this respect, it would be of interest to examine whether homologous *agr* systems of other pathogenic chitinolytic bacteria, such as clostridia and *Enterococcus faecalis* [22], carry out similar regulatory functions in the respective organisms.

Although *agr* may appear as an ideal autoinducer-based sensing system for chitinase induction, our results show that it may only be partly responsible for the induction of the chitinolytic system of *L. monocytogenes*. Indeed, although the production of the two chitinases was decreased in the *agr* mutant, both chitinases were still induced and expressed in considerable amounts in the mutant background. Therefore, other factors that promote chitinase induction in *Listeria* do exist. This is not surprising, given the complexity of the chitinase regulatory system, as well as the fact that chitinase induction is subject to more than one parameter, each of which is likely represented by a different input. Namely, a number of conditions, including chitin availability, as well as the absence of easily fermentable sugars, should all be simultaneously met in order for the chitinases to be fully induced [5], [18] One such condition, monitored by *agr*, could be the retributability/efficiency of macromolecule production.

An open question is whether *agr* is additionally involved in the regulation of the listerial chitinolytic system during infection. The fact that *agr* specifically targets *chiA* opens up this possibility, as *chiA* has been shown to be a virulence factor that downregulates iNOS expression in the host [17]. Since the regulatory pathway behind the induction of *chiA* during infection remains elusive, it would be of interest to examine whether it involves *agr*, especially as *agr* itself has previously been found to be important for pathogenicity [26].

## Supporting Information

Figure S1 **A. Western blot analysis of culture supernatants using an anti-ChiA antibody.** The specificity of the anti-ChiA antibody was confirmed by comparison of wild-type and Δ*chiA* cultures grown at 30 °C in LB+0.05% glucose supplemented with colloidal chitin. Comparison of the wild type to a Δ*chiB* mutant revealed no substantial differences in the production of ChiA. **B. Western blot analysis of culture supernatants using an anti-ChiB antibody.** The specificity of the anti-ChiB antibody was confirmed by comparison of wild-type and Δ*chiB* cultures grown at 30°C in LB+0.05% glucose supplemented with colloidal chitin.
(PDF)
