# Supplementary figures and images for: Chitinase Expression in Listeria monocytogenes Is Positively Regulated by the Agr System
Source: PLoS One. 2014 Apr 21;9(4):e95385. doi: 10.1371/journal.pone.0095385 (PMC3994053; doi:10.1371/journal.pone.0095385)

**A**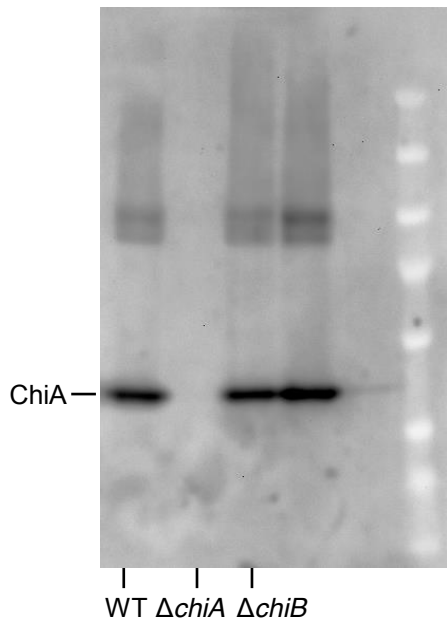**B**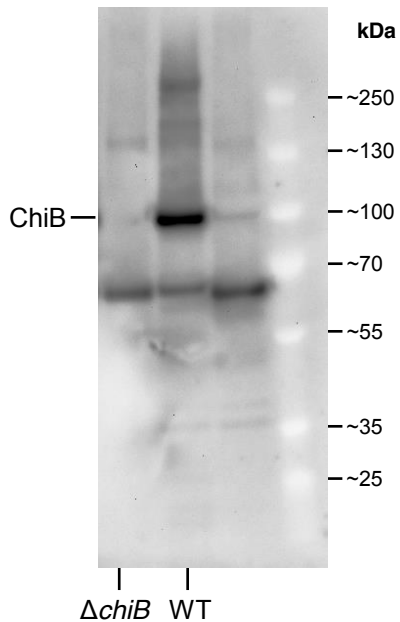

Supplement: Figure S1 — A. Western blot analysis of culture supernatants using an anti-ChiA antibody. The specificity of the anti-ChiA antibody was confirmed by comparison of wild-type and ΔchiA cultures grown at 30 °C in LB+0.05% glucose supplemented with colloidal chitin. Comparison of the wild type to a ΔchiB mutant revealed no substantial differences in the production of ChiA. B. Western blot analysis of culture supernatants using an anti-ChiB antibody. The specificity of the anti-ChiB antibody was confirmed by comparison of wild-type and ΔchiB cultures grown at 30°C in LB+0.05% glucose supplemented with colloidal chitin. (PDF) [file pone.0095385.s001.pdf]
